# Surveillance of Influenza A Virus and Its Subtypes in Migratory Wild Birds of Nepal

**DOI:** 10.1371/journal.pone.0133035

**Published:** 2015-07-15

**Authors:** Dibesh Karmacharya, Sulochana Manandhar, Ajay Sharma, Tarka Bhatta, Pratikshya Adhikari, Adarsh Man Sherchan, Bishwo Shrestha, Manisha Bista, Rajesh Rajbhandari, Mohinder Oberoi, Khadak Bisht, Jean-Marc Hero, Ravi Dissanayake, Maheshwar Dhakal, Jane Hughes, Nitish Debnath

**Affiliations:** 1 Center for Molecular Dynamics Nepal (CMDN), Thapathali-11, Kathmandu, Nepal; 2 Food and agriculture organization, Kathmandu, Nepal; 3 School of Environment, Griffith University, Queensland, Australia; 4 Department of National Parks and Wildlife Conservation, Kathmandu, Nepal; College of Veterinary Medicine, CHINA

## Abstract

Nepal boarders India and China and all three countries lie within the Central Asian Flyway for migratory birds. Novel influenza A H7N9 caused human fatalities in China in 2013. Subclinical infections of influenza A H7N9 in birds and the potential for virus dispersal by migratory birds prompted this study to assess avian H7N9 viral intrusion into Nepal. Surveillance of influenza A virus in migratory birds was implemented in early 2014 with assistance from the Food and Agricultural Organization (FAO). Of 1811 environmental fecal samples collected from seven wetland migratory bird roosting areas, influenza A H9N2 was found in one sample from a ruddy shelduck in Koshi Tappu Wildlife Reserve located in southern Nepal. Avian H7N9 and other highly pathogenic avian influenza viruses were not detected. This study provides baseline data on the status of avian influenza virus in migratory bird populations in Nepal.

## Introduction

Wild birds, particularly aquatic species, are considered natural hosts of avian influenza virus (AIV) [1, 2]. Low pathogenic avian influenza viruses (LPAIV) have co-evolved with their wild host populations to such an extent that infections usually remain subclinical [2]. Wild birds may transmit LPAIV directly or indirectly to poultry, although such infections are generally not sustained [3]. Once introduced into domestic birds, low LPAIV occasionally undergo spontaneous mutations transforming into highly pathogenic avian influenza virus (HPAIV) variants [4, 5].

AIV is a major pathogen of concern to veterinary science, public health, and wildlife conservation sectors. AIV often attains high virulence, transmissibility, and tissue tropism and cover affects a wide host range due to its potential to evolve with capricious genetic shuffling [3]. The HPAIV H5N1 that emerged in late 2003 in Southeast Asia spread throughout East Asia, Southeast Asia, Central Asia, Europe, the Middle East, and Africa, and had a devastating impact on the poultry industry [6, 7]. Ecological and public health concern heightened as H5N1 acquired enhanced virulence and expanded its host range. Thousands of wild migratory birds, over 300 humans, and several other mammals died [8, 9].

The first report of three human fatalities caused by a novel avian influenza H7N9 in China in March 2013 accentuates the fact that avian influenza continues to be an emerging public health threat [10]. Of concern is the fact that the virus produces subclinical infections in domestic and wild birds, dramatically increasing chances of a silent spillover of virus from birds to humans and other animals [11]. Phylogenetic analyses suggest the current circulating novel H7N9 could have originated by acquiring the HA fragment of H7N3 found in ducks, the NA fragment from H7N9 found in migratory birds, and the remaining six internal genes from distinct H9N2 viruses of poultry, gathered in at least two sequential re-assortment steps [10,12–14]. Evidence that H7N9 originated from such intermingling suggests that other combinations of AIV can be expected if close interactions among wild and domestic birds and other species are allowed. Interestingly, 27 genotypes of novel H7N9 have already been identified during recent outbreaks, and additional genotypes are evolving with poultry movement [12].

It is important to have a rigorous avian influenza A surveillance and monitoring mechanism in place to understand the underlying ecological intricacies of the virus and timely capture of circulating novel strain with pandemic potential [6, 15]. Information gathered from such surveillance and monitoring is important in developing a strategic early warning system to rapidly detect HPAIV and identify critical components to mitigate further transmission and spread [15]. Among such components, the role of migratory birds in the introduction and dissemination of AIV cannot be excluded considering the ability of these viral reservoirs to cross countries and continents [6, 16].

Several studies have incriminated migratory birds in the likely spread of HPAIV H5N1from Southeast Asia to the Qinghai Province of China; Mongolia; and Europe [8, 16–18]. If this is true, then, it is even more probable for migratory birds to disperse H7N9 over longer distances owing to its lower pathogenicity in birds. In fact, novel H7N9, genetically similar to the virus isolated from humans and poultry in China, was detected in healthy tree sparrows in Shanghai city in spring 2013 [19]. Systematic AIV surveillance studies of migratory birds have been conducted in Europe [20, 21], North America [16, 22], Africa [23], the Middle East [24], and a few Asian countries [25, 26]. Data on avian influenza is entirely lacking from Nepal, although it lies in a strategically important geographical area between China and India [18]. Understanding this need, an AIV surveillance of the migratory bird population in Nepal was conducted by the Center for Molecular Dynamics Nepal (CMDN) in collaboration with the Food and Agriculture Organization (FAO), Nepal.

**Fig 1:**
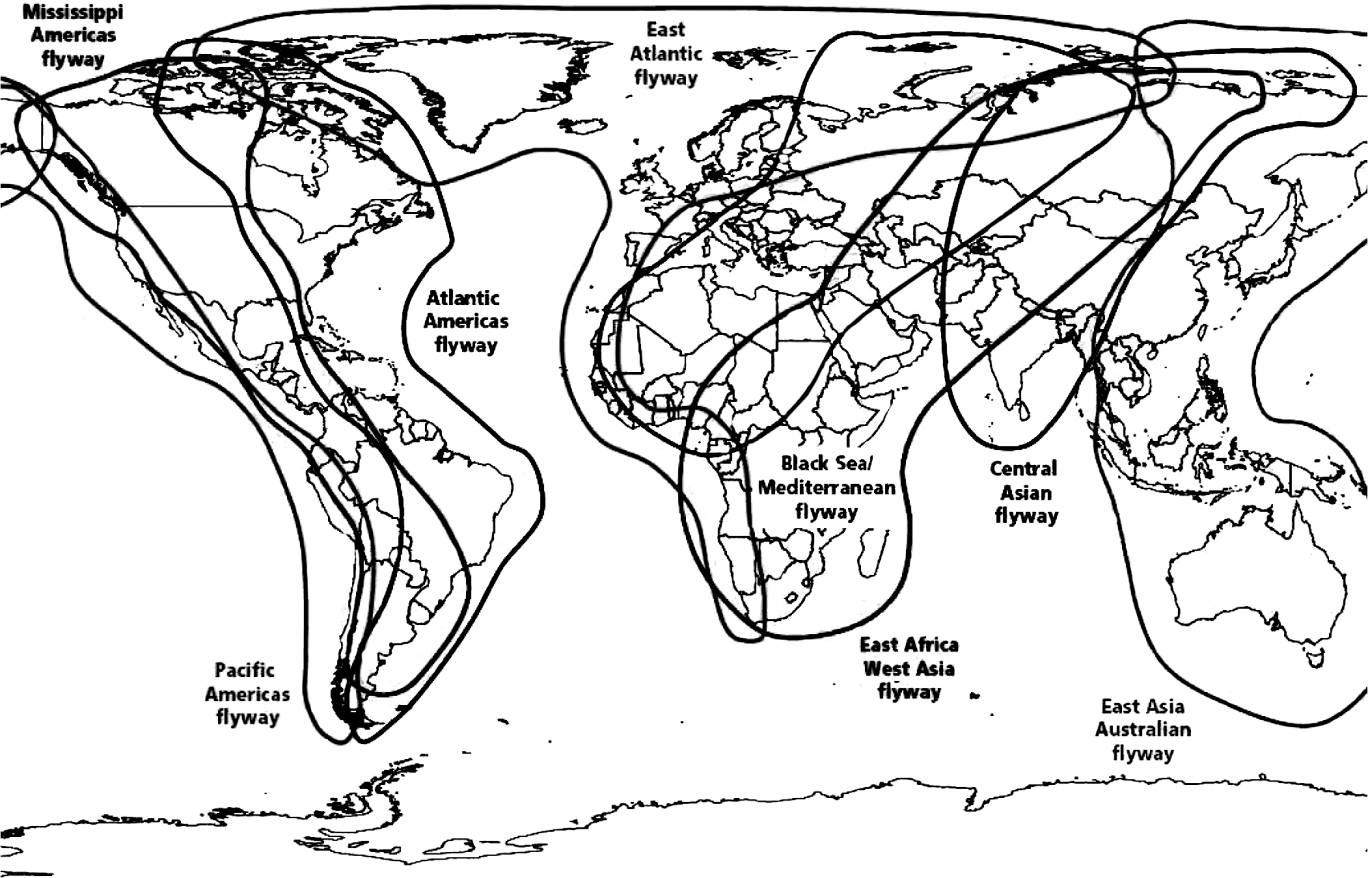
General flyways used by migratory shorebird species. Reprinted from Food and Agriculture Animal Production and Health Manual No. 5[27] under a CC BY license, with permission from FAO copyright unit (2007).

Wetlands in Nepal lie in the path of the Central Asian Flyway (CAF) (Fig 1) [27] and provide over-wintering habitat for a number of migratory bird species [18, 28]. India and China, bordering Nepal, lie along the same flyway and have recurrent histories of H5N1 among poultry and wild birds. The illegal trade of poultry and the movement of poultry products and wild birds across the porous border places Nepal in danger of influenza virus introduction. Since 2009, following trends in neighboring countries, Nepal has suffered perennial H5N1 outbreaks in poultry. Over 234 outbreaks have occurred, causing tremendous damage to the poultry industry [7]. The emergence of the novel anthropogenic H7N9 virus in China and the possibility of its dispersal by migratory birds emphasize the urgency of thorough investigation in Nepal. The main objective of this study was to assess the presence of AIV, particularly H7N9, in the migratory bird population in Nepal.

## Materials and Methods

### Sample size estimation and field sampling

**Fig 2:**
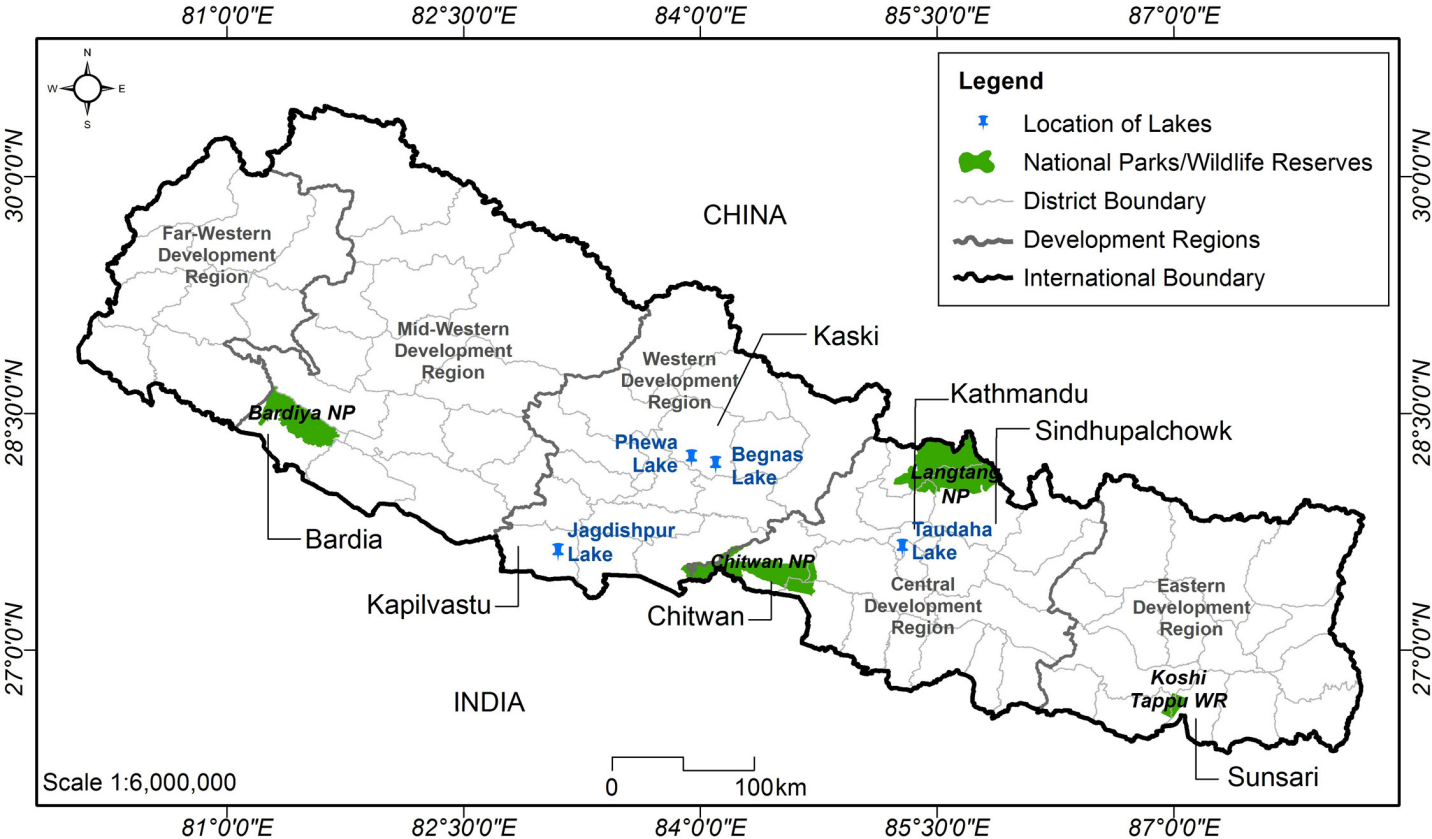
Sampling sites for avian influenza surveillance among wild migratory birds of Nepal.

Seven sites, mostly wetland areas in Nepal that are important stopover and roosting sites for migratory birds, were identified with the assistance of an ornithologist (Fig 2). To guide field researchers and ensure effective sampling, an illustrative field handbook was prepared with key information such as common names, range of habitat and representative images of migratory bird species previously documented in the chosen sampling sites.

Appropriate sample size needed for each sampling site was calculated using the Epi-Tools Survey Toolbox for animal- and herd-level diseases [29]. We considered knowledge from the previous study [30] which found an extremely low H7N9 prevalence of 0.0093% throughout Asia. Sample size was calculated with a precision of 5% for a 95% probability of detecting at least one positive case considering a high sensitivity and specificity rate for RT-PCR assay. A total of 1811 environmental fecal droppings were collected from migratory bird roosting sites between February and March of 2014 (Table 1). Fresh (moist) fecal droppings were collected using a sterile swab, placed in individual cryovials containing sterile Viral Transport Medium [31], and immediately frozen in a liquid nitrogen cryrofreezer. Field data collected included recordings of the GPS locations of the collected samples. Photographs of migratory bird species sighted at the sampling sites were also taken. Upon confirmation of influenza A in any of the collected samples, free ranging backyard poultry (chickens and ducks) were also sampled (June-July, 2014) and screened for influenza A to detect possible viral spillover between wild and local domestic birds. All samples were transported frozen in a dry shipper to CMDN’s Kathmandu-based laboratory and stored in a -80⁰C freezer pending analysis.

**Table 1:** Collection sites and sample sizes for Influenza A virus surveillance of migratory birds in Nepal.

Sampling site	Districts	Number of samples
Chitwan National Park	Chitwan	310
Fewa and Begas lakes	Kaski	294
Taudaha lake	Kathmandu	286
Langtang National Park	Sindhupalchok	250
Jagadishpur lake	Kapilvastu	222
Koshi Tappu Wildlife Reserve	Sunsari	221
Bardia National Park	Bardiya	228
	**Total sample size**	**1811**

### Molecular Diagnosis

#### RNA Extraction, Reverse Transcription, and Real time PCR for H7

Samples were pooled from each site (five samples pooled to one) prior to RNA extraction. RNA was extracted using QIAamp viral RNA mini kit (Qiagen, Germany). Complementary DNA (cDNA) was synthesized immediately after RNA extraction using a SuperScriptFirst-Strand Synthesis Kit (Invitrogen, USA). Positive (strong and moderate) and negative extraction controls provided by Australian Animal Health Laboratory (AAHL) were also extracted and reverse transcribed to obtain cDNA following the same protocol. All extracted RNA was stored at -80⁰C.

All pooled samples were tested for influenza A virus in TaqMan real time PCR targeting viral matrix gene using primers IVA161/IVA162M provided by AAHL [32]. Upon detecting an influenza A positive result in the pooled sample, RNA from the corresponding individual samples was re-extracted and screened for influenza A again to identity the individual sample with influenza A virus. Each assay included a strong positive, a moderate positive, and a negative control, including a no-template control. Individual samples that tested positive for influenza A virus were further characterized for H7 and N9 in separate Taqman real time PCR assays using the primer described by AAHL [33].

#### PCR Characterization of HA and NA Subtypes of Influenza A

Any influenza A matrix gene-positive sample that was negative for H7 was tested for Hemaglutinin (HA) and Neuraminidase (NA) profiling using subtype specific RT-PCR assays with specific primers for HA [34] and NA[35]. The specific viral subtype was identified by DNA sequencing as described below.

#### Bird Species Identification Using DNA Barcoding

The host species of the fecal sample that had influenza A virus was identified using a DNA barcoding assay. The cytochrome oxidase I (COI) region of mitochondrial DNA was amplified using PCR primers (Bird F1 and Bird R1) [36] and the species of the host bird was identified by DNA sequencing as described below.

#### DNA Sequencing of PCR Amplicons

PCR products of expected product size were extracted and purified from agarose gel using a QIAquick Gel Extraction Kit (Qiagen, Germany). The extracted product was DNA sequenced using the Big Dye Terminator Version 3.1 Cycle Sequencing Kit (Applied Biosystems, California, USA) with the same primers used in RT-PCR. The cycle sequenced products were purified with the Big Dye X-terminator Kit (Applied Biosystems, California, USA) and analyzed in an ABI-310 genetic analyzer (Applied Biosystems, California, USA). Sequence ends of raw data were trimmed to reading frame and were used in a BLASTN database search to obtain species identification.

#### Phylogenetic Analysis

Identified H9N2 virus isolates were further characterized to trace their genetic relatedness to other similar viruses. A phylogenetic tree was constructed based on the identified HA sequence data and compared with available H9 sequences in the NCBI database. A bootstrap re-sampling process (1,000 replications) using the neighbor-joining method was employed to assess the robustness of individual phylogeny nodes.

## Results

**Fig 3:**
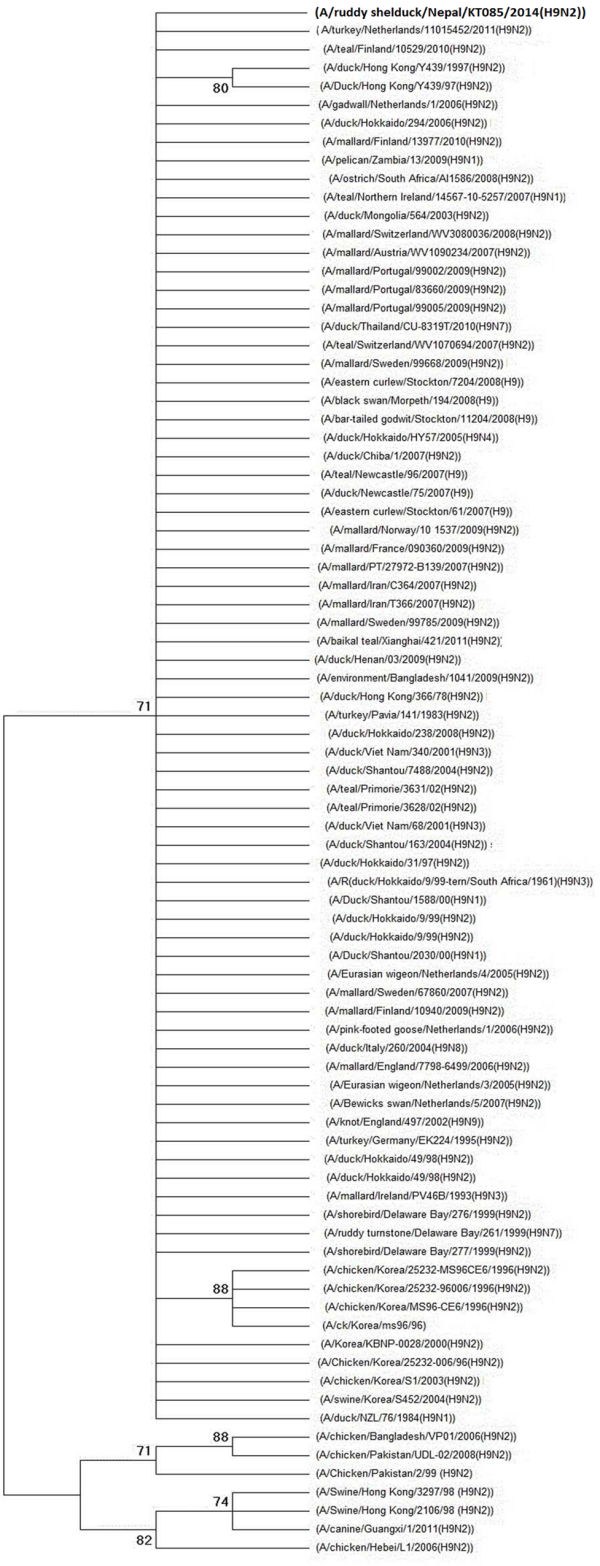
Phylogenetic tree of the HA gene of LPAI H9N2 virus from Nepal. The tree is generated by neighbor-joining method in MEGA 6. Numbers at the branches indicate bootstrap values; only values > 70 are shown.

Of total 1811 samples collected, one (0.055%) tested positive for influenza A virus. This particular sample was collected from the Koshi Tappu Wildlife Reserve (KTWR) in Sunsari district, one of the most important roosting wetland habitats for various migratory bird species. The sample tested negative in the H7N9 QPCR assay. Upon further characterizing HA and NA, the identified influenza A virus was confirmed to be an H9N2 subtype (NCBI Genebank accession number KP830043, http://dx.doi.org/10.6084/m9.figshare.1444228). The phylogenetic tree of the HA gene (Fig 3) of the H9N2 isolate showed clustering to the H9 segment of influenza viruses of various geographical origin isolated from various wild and domestic birds. The host bird for this sample was identified as a ruddy shelduck (*Tadorna ferruginea*) (NCBI Genebank accession number KP718122). No other HPAIV or LPAIV was detected.

The H9N2 positive sample from KTWR was collected close to a human settlement (0.38 km). All cloacal and oro-phyrangeal samples collected from ducks (n = 307) and chickens (n = 103) from households along 5 Km stretch from where influenza A H9N2 positive sample was detected were negative for influenza A virus.

## Discussion

The first cross-sectional molecular surveillance study of avian influenza among migratory birds in Nepal revealed the evidence of presence of the LPAIV H9N2 in a ruddy shelduck, a common migratory bird, close to human settlements in KTWR.

The detection of an infectious AIV in an environmental sample is an important finding with significant public health and wildlife conservation implications. Shedding and persistence of AIV in environment provides the virus with a potential to spill over to sympatric susceptible hosts. AIV is shed in high concentrations in feces [37], and can contaminate water where virus can remain infectious for a prolonged period of time and become a source of spread [38]. Most global AIV surveillance studies have used invasive samples (cloacal or oro-pharyngeal swabs). Wild birds are known to be natural hosts; detecting virus in invasive samples simply represents virus existing in vivo. While such studies are important in understanding the temporal and spatial diversity of circulating viral pool, their practical implication might not be as significant as finding a potent virus that has been shed into the environment where it may infect other hosts or impact pathogen spillover.

Poultry farming is important to Nepal’s economy, contributing nearly 4% of its total gross domestic products (GDP). Free-ranging domesticated birds like chickens and ducks are popular in rural Nepal. Since the first report of H5N1 among backyard chickens in the eastern district of India bordering West Bengal in January 2009, Nepal has experienced perennial outbreaks of H5N1 in poultry, in both back-yard and commercial flocks, incurring huge economic losses [7, 39]. Wetlands and wildlife reserves of Nepal are the ideal home for many wintering and summering wild migratory birds following the CAF [28]. Wildlife habitat is increasingly encroached by human settlements in Nepal due to rapid population growth and the limited availability of farm land. Such dramatic changes in landscape and landmass use increase the chances of interspecies interactions, often providing a “mixing-vessel” conducive for the emergence and spillover of novel zoonotic pathogens like AIV. Open cultivated fields in the vicinity of wildlife protected buffer zones are a common attraction point for both domestic and wild bird species as a source of food (grains and worms released during field tilling). Furthermore, ponds and lakes are often shared by both wild and domestic waterfowl, increasing the possibility of viral spillover from wild to domestic birds and vice versa. Although we didn’t detect any AIV in domestic bird samples, this does not entirely exclude the possibility of viral presence in them.

The ruddy shelduck is an important migratory bird species of the CAF and a common winter visitor to Nepal [28]. Previous studies [18, 40] have shown that movement of ruddy shelduck is strongly associated with the spatial-temporal pattern of AIV outbreaks in poultry in South Asia. This bird species was reported to carry H5N1 in the 2005 outbreak at the Qinghai Lake of China [8]; LPAIV was isolated as in other studies [25]. This corroborates the carrier role of this long-range migratory waterfowl species and the subsequent need to consider ducks as one of the sentinel species in AIV surveillance. A phylogenetic study revealed the clustering together of clade 2.3.2.1 of H5N1viruses originating from poultry in Nepal, Bangladesh, and India indicating active movement of virus across neighboring porous borders, for which the crow (*Corvus splendens*) has been incriminated[26]. Detection of the same clade of H5N1 virus in crows from various areas of Nepal, including near the Indian border in 2012 and 2013 [7] provides evidence of the reciprocation of virus between wild and domestic birds in Nepal. The phylogenetic clustering of the identified H9 segment to that of other prevalent AIV isolated from wild and domestic birds of mixed geographical origin suggest a possible role of migratory birds in worldwide viral spread.

In a 2-month study, we detected AIV in one of 1811samples by RT-PCR, yielding an approximate AIV detection rate of 0.055% among migratory birds in Nepal. The prevalence of AIV in migratory birds has been markedly different among various studies. The prevalence has ranged from 2.6% in an 8-year European study with 36,809 samples [21], 1.7% in a 10-month study in Alaska [22], 3% in a 4-year Iranian study with 1146 sample [24], 3.5% in a 3-month African study with 4553 samples [25], to 0% in 10,788 samples collected in Bangladesh [26].

The AIV detection rate in our study appears considerably lower than in other studies with exception of the Bangladesh study. The brief sampling period of 2 months may be one obvious explanation for the low AIV detection in the migratory birds in Nepal. In addition, this study adopted non- invasively collected fecal droppings as the environmental sample source to avoid additional financial, technical, and logistical needs required for collecting invasive samples such as oro-pharyngeal or cloacal swabs from wild birds [15]. While environmental samples were convenient alternative, influenza viruses are enveloped RNA viruses that are fragile and prone to easily disintegrate when exposed to elements such as sunlight, desiccation, extreme temperatures, and fungal growth, making them challenging to detect using molecular methods. Lengthening the sampling period, increasing the sample size, and collecting some invasive samples might have increased our AIV detection rate.

In our study, we did not detect H7N9 or HPAI including H5N1. Low HPAI detection is a common finding of most AIV surveillance studies among healthy wild migratory birds. No HPAIV was detected in large surveillance studies conducted in the U.S. [16], Europe [20, 21], Alaska [22], Africa [23], Iran [24], and Mongolia [25]. This indicates that HPAIV is rare among healthy wild bird populations [3], which could be due to greater tissue tropism of HPAIV for the upper respiratory tract than for the gastrointestinal tract as evidenced by experimental studies [41].

The one AIV positive wild sample in this study was H9N2, a low pathogenic subtype. This subtype has occasionally been detected among wild bird species in North America, Europe, and Iran. Unlike other LPAIV, which soon disappear after a brief circulation in poultry, H9N2 has succeeded in attaining an endemic status among poultry, mostly chickens, in most parts of the world [3]. Hence, poultry carrying the H9N2 subtype have been critically observed for their incubator role in the evolution of the currently circulating novel anthropogenic subtypes H7N9 and H10N8, both of which have internal genes originating from H9N2 [42, 43]. It has been postulated that H9N2-derived internal genes enabled the novel H7N9 to evolve and will facilitate any migratory-bird-derived LAIV subtype to persist among poultry and be transmitted to humans [42].

## Conclusions

This was the first AIV surveillance study on wild migratory birds in Nepal and provides strong evidence of the potential for AIV spillover through migratory birds in the region. A long-term, continuous, and rigorous seasonal surveillance of wild birds at various sentinel sites is recommended to formulate a complete picture of the temporal, spatial, and strain variations of AIV in Nepal.
