# Correction: Surveillance of Influenza A Virus and Its Subtypes in Migratory Wild Birds of Nepal

**DOI:** 10.1371/journal.pone.0218344

**Published:** 2019-06-06

**Authors:** Dibesh Karmacharya, Sulochana Manandhar, Ajay Sharma, Tarka Bhatta, Pratikshya Adhikari, Adarsh Man Sherchan, Bishwo Shrestha, Manisha Bista, Rajesh Rajbhandari, Mohinder Oberoi, Khadak Bisht, Jean-Marc Hero, Ravi Dissanayake, Maheshwar Dhakal, Jane Hughes, Nitish Debnath

There is an error in the caption for Fig 1, “General flyways used by migratory shorebird species”. Please see the complete, correct Fig 1 caption here.

**Fig 1:**
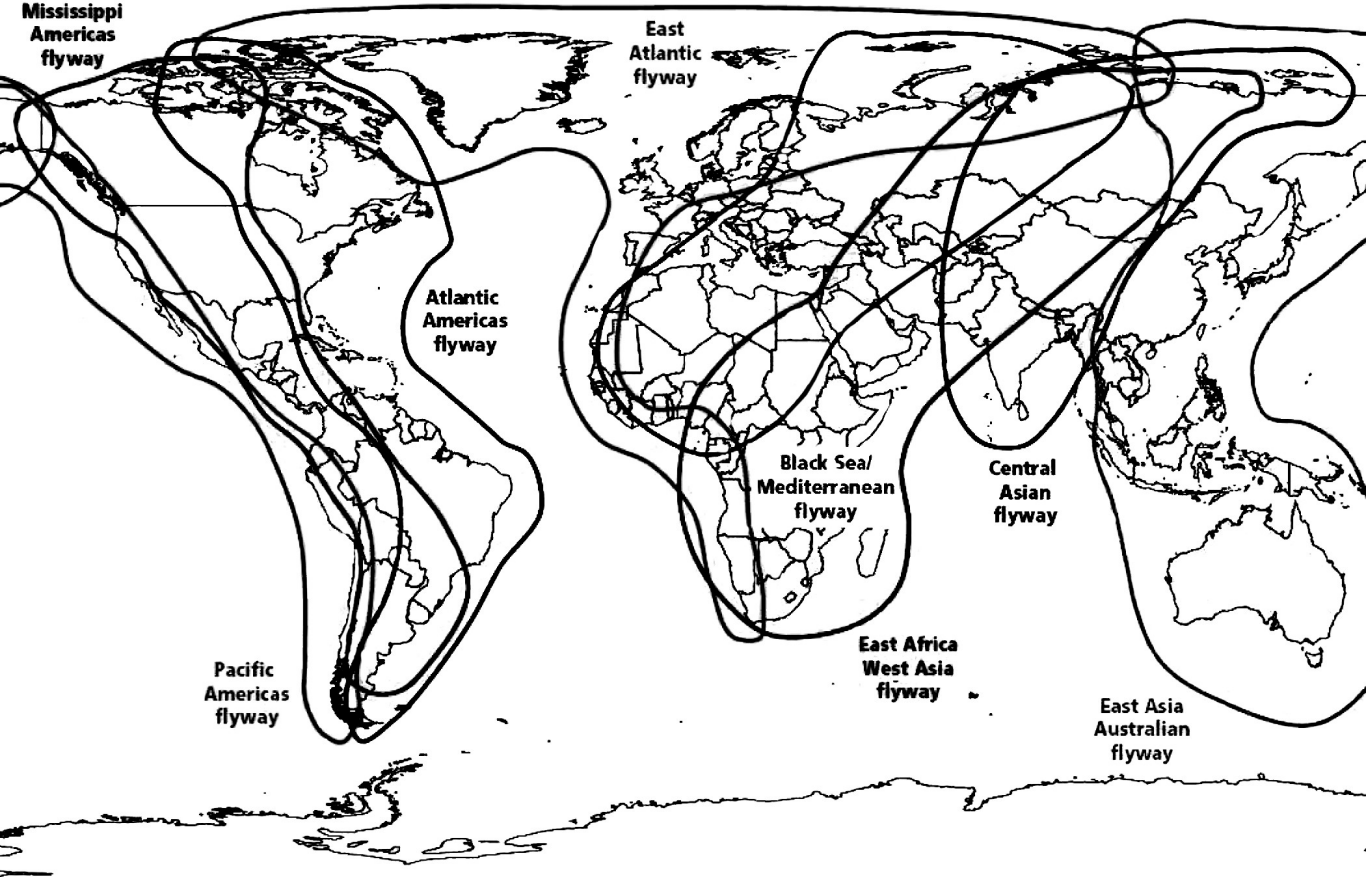
General flyways used by migratory shorebird species. Reprinted from Food and Agriculture Animal Production and Health Manual No. 5 [27]. Copyright Food and Agriculture Organization of the United Nations. Reproduced with permission.

Additionally, the authors provide clarifications to the conclusions and additional phylogenetic analysis as follows:

The Discussion section states, “The detection of an infectious AIV in an environmental sample is an important finding with significant public health and wildlife conservation implications.” Here the authors clarify that in this study RNA from a potentially viable low pathogenicity H9N2 influenza A virus was detected in an environmental fecal sample; however, the findings in this study do not formally demonstrate that the detected virus was infectious in the environment.

The Discussion also states, “The phylogenetic clustering of the identified H9 segment to that of other prevalent AIV isolated from wild and domestic birds of mixed geographical origin suggest a possible role of migratory birds in worldwide viral spread.” Here the authors clarify that this statement should be considered speculative, as the phylogenetic tree in Fig 3 presents unresolved polytomies, and as such, does not allow for conclusions to be drawn regarding the relationships between the lineages and the role of migratory birds in worldwide spread. The obtained sequence size in the study was 466 bp, and this limited data does not allow for detailed analyses. An improved phylogenetic analysis has been carried out; the methodology and results of this analysis are provided below as Supporting Information (S1 File, S2 File). Inferences are made regarding the grouping under clades and the consistency of this evidence with migratory spread, but this discussion is speculative as statistical support for these inferences is limited.

## Supporting information

S1 File Updated Phylogenetic Analysis. (XLSX)

S2 File Updated Phylogenetic Tree. (JPG)
